# An analysis of socio-demographic patterns in child malnutrition trends using Ghana demographic and health survey data in the period 1993–2008

**DOI:** 10.1186/1471-2458-13-960

**Published:** 2013-10-16

**Authors:** Dickson A Amugsi, Maurice B Mittelmark, Anna Lartey

**Affiliations:** 1 Department of Health Promotion and Development, Faculty of Psychology, University of Bergen, Christiesgt. 13, 5020 Bergen, Norway; 2 Department of Nutrition and Food Science, University of Ghana, Legon, Accra, Ghana

**Keywords:** Malnutrition, Children under-five, Socio-demographic factors, Ghana

## Abstract

**Background:**

A small but growing body of research indicates that progress in reducing child malnutrition is substantially uneven from place to place, even down to the district level within countries. Yet child malnutrition prevalence and trend estimates available for public health planning are mostly available only at the level of global regions and/or at country level. To support carefully targeted intervention to reduce child malnutrition, public health planners and policy-makers require access to more refined prevalence data and trend analyses than are presently available. Responding to this need in Ghana, this report presents trends in child malnutrition prevalence in socio-demographic groups within the country’s geographic regions.

**Methods:**

The study uses the Ghana Demographic and Health Surveys (GDHS) data. The GDHS are nationally representative cross-sectional surveys that have been carried out in many developing countries. These surveys constitute one of the richest sources of information currently available to examine time trends in child malnutrition. Data from four surveys were used for the analysis: 1993, 1998, 2003 and 2008.

**Results:**

The results show statistically significant declining trends at the national level for stunting (F (1, 7204) = 7.89, p ≤ .005), underweight (F (1, 7441) = 44.87, p ≤ .001) and wasting (F (1, 7130) = 6.19, p ≤ .013). However, analyses of the sex-specific trends revealed that the declining trends in stunting and wasting were significant among males but not among females. In contrast to the national trend, there were significantly increasing trends in stunting for males (F (1, 2004) = 3.92, p ≤ .048) and females (F (1, 2004) = 4.34, p ≤ .037) whose mothers had higher than primary education, while the trends decreased significantly for males and females whose mothers had no education.

**Conclusions:**

At the national level in Ghana, child malnutrition is significantly declining. However, the aggregate national trend masks important deviations in certain socio-demographic segments, including worsening levels of malnutrition. This paper shows the importance of disaggregated analyses of national child malnutrition data, to unmask underlying geographic and socio-demographic differences.

## Background

Child malnutrition is a long-standing and continuing public health problem in many regions of the world, despite global-level progress in the reduction of this problem during the past two decades [1]. Globally, it is estimated that childhood stunting (short stature for age) reduced from 34% to 27% and underweight from 27% to 22%, between 1990 and 2000 [1] and stunting is predicted to reduce to 22% by the next decade [2]. However, evidence shows that global estimates cannot be used to monitor progress at the regional level [3]. In Africa, the prevalence of stunting declined marginally from 40.5% in 1980 to 35.2% in 2000 [3], and between 1990 and 2010 the prevalence of childhood stunting in Africa stagnated at about 40% (2). If this trend continues, we cannot expect significant improvement in the next decade. Global estimates are not good indicators of regional trends, and it can be misleading to use regional-level data to estimate the magnitude and trend in malnutrition at the sub-regional level. During the same period when stunting decreased in North Africa, it actually increased in Eastern Africa, while Western Africa showed very little change [3].

The lesson about the limits to generalizability of large area data to smaller areas is pertinent also to intra-national demographic divisions such as urban/rural, Regions, States and Districts. In 2008 World Health Organization (WHO) estimates of the prevalence of stunting in Ghana, the urban rate was 22% while the rural rate was 33%; the Region with the lowest rate was Greater Accra with 17% while the Eastern region had the highest rate of 39%, more than two-fold difference [4].

Aside from geographic variation, child malnutrition prevalence varies substantially by socio-demographic factors such as the child’s age and sex [5-8]. Therefore, in order to study trends in malnutrition at a level useful to public health planning, analyses need to be stratified by defined socio-demographic segments; overall trends may mask important departures from the general trend.

It is also vital that malnutrition be defined consistently over the time that a trend analysis is undertaken, and this has been challenging in the area of child malnutrition. The National Centre for Health Statistics (NCHS) international growth reference, that had been in use by WHO since 1978, were changed in 2006. Therefore, the published estimates before and after 2006 are not comparable, except for studies that have used the old standards also in the analysis of data generated since 2006. This strategy results in consistency [9,10].

Health ministries, as well as governmental and non-governmental health and development agencies, need access to new analyses of child malnutrition and long-term trends, that use the new WHO growth standards exclusively, and that provide disaggregated estimates for defined socio-demographic segments of the under-five population.

Turning to the socio-demographic factors that influence child under-nutrition, there is a vast theoretical and empirical literature advancing hypotheses and data on the link between socio-demographic factors and health generally and child health in particular [11-13]; a review of that literature is well beyond the scope of this paper. However, below is a detail description of various factors that might influence the nutritional status of children under five years.

Child age and gender are of such fundamental importance to the study of malnutrition time trends that they should not be ignored. Normal child growth and malnutrition are defined by WHO growth standards separately for boys and girls and for age bands separating major phases of development such as pre- and post-weaning. Yet many, if not most published studies, tend to group boys and girls and/or fail to undertake age-specific analyses. When age- and gender-specific analyses are undertaken, they show consistent differences in malnutrition prevalence that would have been masked by less-differentiated analyses [5-8].

Furthermore, a consistent finding in child malnutrition studies is that children who reside in rural areas have higher rates of stunting and underweight than those in urban areas [6-8,14,15], while the pattern for wasting is somewhat inconsistent [16]. Even when controlling for poverty level, malnutrition prevalence is always lower in urban areas [17] than in rural area. Aside from urban/rural differences, significant intra-country regional variation in child malnutrition has been documented [6,7]. In Ghana, the regions with the greatest burden of child malnutrition are those in the northern part of the country [5]. De Poel and colleagues [5] point out that the regional patterns observed in Ghana reflect ecological constraints, worse general living conditions, and poorer access to public facilities in the Northern regions. There is also an intergenerational aspect, with Northern women who suffered from malnutrition when they were children being more likely to give birth to children with low birth weight [5,18], placing such children at risk of being malnourished [19]. As for urban/rural trends, new analyses are needed to track regional time trends in malnutrition.

One of the most robust findings in the public health literature is that health is associated with socio-economic status in a graded way. Increments in household material standards (as measured for example by the Wealth Index) are associated with increments in health [20], including child nutritional status [8,21,22]. Children from wealthy households are better off compared to children from poor households. In Ghana for instance, children in the poorest households are at more than twice the risk of being stunted compared to those from the richest households [23]. Hence, prevalence and trend studies of child malnutrition should differentiate among socio-economic status groups.

Additionally, the level of maternal education is consistently, although sometimes only weakly, associated with child malnutrition [24-26].

As the time when an accounting of progress on development goals for the Global South is coming due – especially the Millennium Development Goals in 2015—planners and policy makers need reliable and valid information on child malnutrition trends that are sufficiently detailed in their socio-demographic breakdown to illuminate where goals are being met, and where challenges remain. The needed data exist, gathered in four Ghana Health and Demographic Surveys spanning 15 years from 1993 to 2008. However, these data require reanalysis, using current WHO Child Growth Standards and providing breakdowns by key socio-demographic segments.

The aims of this paper are: [1] to present findings on national child malnutrition prevalence trends in Ghana in the period 1993 to 2008; [2] to disaggregate the national level data to socio-demographic and geographic sub-groups, and [3] to compare and contrast the national level findings on malnutrition with analysis based on the sub-group.

## Methods

### Data sources

This study uses data from the Ghana Demographic and Health Surveys [27]. The surveys were conducted in 1993, 1998, 2003 and 2008 by the Ghana Statistical Service and Ghana Health Service, with technical and financial support from ICF Macro through the USAID-funded MEASURE DHS programme. The surveys were designed to be representative at the national, regional and rural–urban levels. A two-stage probabilistic sampling design was used to select clusters (census districts) at the first stage. The second stage involved the selection of households from these clusters. All women and men aged 15–49 in the selected households were eligible to participate in the surveys. The household response rates were 98.4% in 1993, 99.1% in 1998, 98.7% 2003, and 98.9% in 2008. The data were collected at two levels—the household and individual levels. At the household level, information was collected on household characteristics such as source of drinking water, toilet facilities, cooking fuel, and assets of the household. At the individual level, questionnaires were administered to women aged 15–49 and men aged 15–59 to gather information on individual characteristics and health behaviors, and information on children in the household.

### Variables

#### Nutritional Status of Children

Child nutritional status was assessed by height-for-age z-scores, weight-for-height z-scores and weight-for-age z-scores using the new WHO Child Growth Standards [28]. A child was considered stunted, wasted or underweight if their height-for-age, weight-for-height or weight-for-age z-scores were further than −2 standard deviations from the median of the reference sample used to construct the WHO 2006 growth standards.

The DHS 2008 survey used the new WHO Child Growth Standards [29], while the earlier DHS surveys used the NCHS growth reference [30-32]. For the purposes of cross-survey comparability, we calculated z-scores using the new WHO Child Growth Standards, using a syntax file provided by the WHO [33]. This syntax file automatically flagged all biological implausible values. Thus, height-for-age z-scores less than −6.0 and greater than +6.0, weight-for-height z-scores less than −5.0 and greater +5.0 and weight-for-age z-scores less than −6.0 and greater than +5.0 are excluded from our analysis.

### Socio-demographic variables

The socio-demographic variables included child sex and age, mother’s education, urban/rural residence, region of residence and Wealth Index (composed using factor analysis to combine household-level information about housing quality and ownership/access to material goods).

Some of the variables were re-coded in order to attain reasonable sample sizes, and also based on suggestions in the literature. For maternal education, incomplete and complete primary were recoded as “Primary”, and incomplete secondary, complete secondary and tertiary as “Some high school or higher”. The region variable was recoded into five categories—Upper East and West regions as “Upper”, Ashanti and Brong Ahafo regions as “Middle”, Western, Central, Volta and Eastern regions as “South” while Greater Accra and Northern regions remained “Accra” and “Northern” respectively [34].

### Data analysis

We used SPSS for windows version 19.0 to perform the data analysis. Using the definitions described above, children were classified as stunted/not stunted, wasted/not wasted and underweight/not underweight. All analyses were stratified by sex. We used the Chi Square test for homogeneity to calculate the confidence intervals for prevalence estimates, which are reported in the Tables. We used logistic regression to test the significant of trends over time. The results of these tests are given in the text only (and not in the Tables). A trend was considered statistically significant if the p-value was less than 0.05. Since the DHS sampling design includes both under- and over-sampling, all analyses were conducted with sample-weighted data. The weights also accounted for non-response. It is possible to use multi-level methods to adjust for cluster-level design effect. This should be done in analyses that are sensitive to within-census district social commonalities. We have not adjusted for the possible design effect of cluster, due to the implausibility that census district is an important source of dependency in the child growth data. This strategy avoids over-adjustment of the analyses.

### Ethics

The DHS project sought and obtained the necessary ethical approvals from ethics committees in Ghana before the surveys were carried out. Informed consent was obtained from study participants before they were allowed to participate in the surveys. The survey data sets used in this report were completely anonymous with regard to participant identity.

## Results

### Participation rates

Anthropometric data (weight and length/height) were collected from children 0–60 months in the surveys conducted in 1998, 2003, and 2008, and from children 0–36 months in the 1993 survey. To make cross-year comparability possible, we restricted our study sample to children less than 3 years old. Of 2,204 children who were part of the 1993 survey, anthropometry data were available for 1,966 (89.2%), and of the 2,067 children who were part of the 1998 survey, anthropometry data were reported for 1,778 (86.0%). In the 2003 survey, of the 2,439 children in the study anthropometry data were available for 1,933 (79.3%). In 2008, 1,904 children participated and anthropometry data were available for 1,558 (81.8%).

### Trends in stunting (height-for-age)

Table 1 shows the national prevalence of stunting, underweight and wasting over the study period, in total and separately for males and females, and the results of Chi Square tests of homogeneity. The tests for homogeneity are statistically significant (except for the test for wasting for females), indicating that the prevalence estimates changed significantly over the years (the Chi Square tests in Tables 2, 3, 4, 5, 6, 7, 8, 9, 10, 11, 12, 13, 14, 15, 16 and 17 are also tests of homogeneity). Tests for linear trends were undertaken using logistic regression; all F statistics and associated probabilities reported in this text were calculated using logistic regression. The declining trend in total stunting was statistically significant (F (1, 7204) = 7.89, p ≤ .005). This was also the case for underweight (F (1, 7441) = 44.87, p ≤ .001) and wasting (F (1, 7130) = 6.19, p ≤ .013). However, the analyses of the sex-specific trends revealed that the declining trend in stunting was significant only among males (F (1, 7204) = 5.79, p ≤ .016) as was the trend for wasting (F (1, 7130) = 6.56, p ≤ .010). The declining trends in underweight were statistically significant for both males (F (1, 7441) = 26.69, p ≤ .001) and females (F (1, 7441) = 20.14, p ≤ .001).

**Table 1:** Prevalence and 95% confidence intervals (C.I.) in childhood malnutrition at the national level, ages 0 through 36 Months, 1993 to 2008

	**1993**		**1998**		**2003**		**2008**				
**Variables**	**%**	**C.I.**	**%**	**C.I.**	**%**	**C.I.**	**%**	**C.I.**	**p**	***X***^***2***^	**Total N**
***Stunting***	***33,5***	***31.4, 35.6***	***25,6***	***23.5, 27.7***	***33,6***	***31.3, 35.9***	***25,7***	***23.4, 28.2***	***0,001***	***51,472***	***7205***
Male	37.2	34.3, 40.3	28.4	25.4, 31.6	37.4	34.2, 40.7	29.2	25.8, 32.9	0.001	29.422	3603
Female	29.5	26.7, 32.5	22.9	20.1, 25.8	29.8	26.8, 33.0	22.2	19.2, 25.6	0.001	23.178	3602
***Underweight***	***25,1***	***23.2, 27.0***	***19,8***	***17.9, 21.7***	***20.0***	***18.2, 21.9***	***15,1***	***13.3, 17.1***	***0,001***	***54,863***	***7442***
Male	27.0	24.3, 29.8	19.7	17.1, 22.5	22.6	20.0, 25.5	16.9	14.3, 20.0	0.001	30.072	3733
Female	23.1	20.5, 25.8	19.9	17.3, 22.7	17.4	15.1, 20.0	13.2	10.9, 15.8	0.001	30.582	3709
***Wasting***	***14,6***	***13.1, 16.2***	***13,3***	***11.7, 15.0***	***11.0***	***9.6, 12.6***	***12,4***	***10.7, 14.3***	***0.010***	***11,317***	***7131***
Male	15.5	13.4, 18.0	15.0	12.7, 17.7	11.3	9.3, 13.6	13.3	10.8, 16.3	0.051	8.741	3556
Female	13.6	11.6, 15.9	11.6	9.6, 14.0	10.7	8.9, 13.0	11.4	9.3, 14.0	0.281	4.121	3575

**Table 2:** Prevalence and 95% confidence intervals (C.I.) in stunting among Ghanaian children ages 0 to 36 months, by age and sex, 1993 to 2008

	**1993**		**1998**		**2003**		**2008**				
**Variables**	**%**	**C.I**	**%**	**C.I**	**%**	**C.I**	**%**	**C.I**	**p**	***X***^***2***^	**Total N**
**Age**											
***0-5***											
Male	15.0	10.5, 21.0	7,5	4.4, 12.6	15,9	10.8, 22.8	9.1	4.7, 17.0	0,058	7.467	633
Female	12.8	8.8, 18.2	8.3	4.7, 14.1	11.0	6.8, 17.3	5.2	2.4, 11.0	0,139	5.486	626
***6-11***											
Male	16.0	11.4, 22.1	12.2	7.7, 18.7	23.7	17.3, 31.5	16.9	11.2, 24.6	0,062	7.350	635
Female	12.1	7.9, 18.1	12.8	8.4, 19.0	13.9	9.7, 19.6	11.5	6.7, 19.1	0,923	.482	673
***12-23***											
Male	45.4	40.1, 50.7	35.8	30.6, 41.5	43,2	37.7, 48.8	37.0	30.7, 48.8	0.040	8.320	1267
Female	32.3	27.2, 37.9	25.4	20.7, 30.8	34,2	28.8, 40.0	29.3	23.9, 35.5	0,096	6.351	1199
***24-35***											
Male	55.1	49.2, 60.7	40.4	34.2, 46.8	49,9	43.7, 56.0	38.4	32.0, 45.3	0,001	19.453	1068
Female	47.6	42.0, 53.3	34.5	28.8, 40.7	44,3	38.4, 50.4	29.1	23.3, 35.8	0,001	23.155	1104

**Table 3:** Prevalence in stunting among Ghanaian children by place of residence, 0-36 Months 1993 to 2008 with 95% CI

	**1993**		**1998**		**2003**		**2008**				
	**%**	**C.I**	**%**	**C.I**	**%**	**C.I**	**%**	**C.I**	**p**	***X***^**2**^	**Total N**
**Residence**											
***Urban***											
Male	26.4	21.6, 31.9	22.5	17.2, 28.8	29.5	24.2, 35.5	25.9	24.2, 35.5	0,314	3,552	1024
Female	16.6	12.5, 21.6	14.4	10.0, 20.4	22.0	16.9, 27.9	18.7	14.2, 24.4	0,142	5,452	977
***Rural***											
Male	41.5	37.9, 45.2	30.5	27.0, 34.3	41,5	37.6, 45.5	31.1	26.8, 35.7	0,001	29.329	2579
Female	34.4	30.9, 38.0	25.5	22.2, 29.0	33,6	30.0, 37.4	24.4	20.6, 28.7	0,001	23,658	2625

**Table 4:** Trends in stunting among Ghanaian children by region, 0-36 Months 1993 to 2008 with 95% CI

	**1993**		**1998**		**2003**		**2008**				
	**%**	**C.I**	**%**	**C.I**	**%**	**C.I**	**%**	**C.I**	**p**	***X***^**2**^	**Total N**
**Region**											
***Upper***											
Male	36.8	27.8, 47.0	37.3	30.0, 45.2	40,9	31.7, 50.6	26.4	18.8, 35.8	0,331	3.423	550
Female	33.9	25.7, 43.3	28.5	22.0, 36.1	26,7	19.6, 35.2	35.1	25.8, 45.7	0,662	1,588	541
***Middle***											
Male	40.0	34.4, 45.9	28.8	22.7, 35.7	35,1	29.3, 41.3	26.4	20.5,33.4	0,006	12.533	926
Female	30.6	25.3 , 36.5	21.3	16.1, 27.7	34.0	28.2, 40.4	22.3	16.8,29.0	0,003	14.227	918
***South***											
Male	35.3	30.7, 40.1	27.4	22.9, 32.3	35,2	29.8, 40.9	31.5	25.7, 37.9	0,059	7.452	1312
Female	28.6	24.3, 33.2	23.5	19.5, 28.1	25.1	20.6, 30.3	23.0	18.1, 29.7	0,283	3.807	1378
***Accra***											
Male	22.3	15.0, 31.9	21.7	14.1, 31.8	21.9	14.1, 32.3	24.0	13.8, 38.3	0,985	.151	324
Female	14.8	8.8, 23.8	12.2	6.7, 21.3	14,2	7.9, 24.1	14.3	7.8,25.3	0,965	.272	324
***Northern***											
Male	50.0	40.9, 59.1	34.2	25.2, 44.6	58,9	50.7, 66.5	33.8	25.8, 43.0	0,001	19.648	491
Female	39.2	30.0, 49.2	35.9	26.0, 47.1	46,8	38.8, 55.0	20.4	13.5, 29.5	0,001	17.451	441

**Table 5:** Trends in stunting among Ghanaian children by maternal education, 0-36 Months 1993 to 2008 with 95% CI

	**1993**		**1998**		**2003**		**2008**				
	**%**	**C.I**	**%**	**C.I**	**%**	**C.I**	**%**	**C.I**	**p**	***X***^**2**^	**Total N**
**Maternal education**											
***No education***											
Male	43.9	38.9, 48.9	32.3	27.5, 37.5	41,8	36.9, 47.0	28.6	23.1, 34.8	0,001	21.362	1502
Female	33.2	28.7, 38.1	27.4	22.9, 32.4	37,4	32.6, 42.4	19.9	15.2, 25.7	0,001	22.930	1498
***Primary***											
Male	35.1	31.2, 39.2	33.1	26.4, 40.5	33,9	27.4, 41.1	34.7	27.8, 42.4	0,963	.284	1125
Female	28.7	25.0, 32.8	22.3	16.5, 29.4	22.6	17.3, 29.0	24.6	18.2, 32.3	0.180	4.885	1073
***Some high school+***											
Male	15.3	8.1, 26.8	22.1	17.8, 27.2	34,9	29.6, 40.6	26.2	21.0, 32.1	0,001	19.950	975
Female	9.6	4.1, 21.1	19.5	15.6, 24.1	26,2	21.2, 31.8	22.7	18.3, 27.8	0.020	9.867	1030

**Table 6:** Trends in stunting among Ghanaian children stratified by maternal education and place of residence, 0-36 Months 1993 to 2008 with 95% CI

	**1993**		**1998**		**2003**		**2008**				
	**%**	**C.I**	**%**	**C.I**	**%**	**C.I**	**%**	**C.I**	**p**	***X***^**2**^	**Total N**
**Urban**											
***No education***											
male	35.7	24.3, 49.0	33.0	21.7, 46.6	32.1	21.7, 44.6	25.4	15.5, 38.8	.69	1.60	252
female	22.0	12.6, 35.6	17.9	8.5, 33.8	35.2	22.9, 49.9	21.6	10.6, 39.1	.23	4.61	177
***Primary***											
male	26.1	20.2, 33.2	26.9	16.4, 40.9	22.0	13.4, 33.8	31.3	20.4, 44.7	.70	1.58	361
female	18.1	13.0, 24.7	17.5	7.9, 34.3	13.0	5.9, 26.3	24.2	14.1, 38.3	.56	2.31	310
***High school+***											
male	12.2	5.6, 24.7	12.2	7.3, 19.6	28.8	22.3, 36.4	22.2	15.7, 29.9	.004	14.29	496
female	8.1	2.6, 22.4	15.5	9.5, 24.2	21.4	14.3, 30.8	16.5	10.9, 24.2	.31	3.90	405
**Rural**											
***No education***											
male	43.1	37.8, 48.6	30.4	25.7, 35.5	40.8	36.1, 45.8	26.8	21.3, 33.1	.001	27.76	1467
female	39.9	31.9, 42.1	30.5	25.0, 36.5	41.5	35.5, 47.7	21.1	15.4, 28.3	.001	22.28	1104
***Primary***											
male	38.0	33.3, 43.1	33.1	25.9, 41.1	35.9	28.5, 44.1	32.8	25.1, 41.5	.63	1.91	842
female	35.5	30.6, 40.8	24.1	16.7, 33.5	28.7	21.5, 37.3	28.0	19.5, 38.4	.11	6.51	685
***High school+***											
male	20.0	5.0, 54.2	26.7	21.4, 32.8	37.0	30.1, 44.4	27.6	21.0, 35.4	.09	6.66	611
female	20.0	6.6, 47.1	21.5	16.3, 27.9	33.5	25.8, 42.1	31.3	23.8, 39.8	.06	7.52	493

**Table 7:** Trends in stunting among Ghanaian children by household wealth quintile, 0-36 Months 1993 to 2008 with 95% CI

	**1993**		**1998**		**2003**		**2008**				
**%**	**%**	**C.I**	**%**	**C.I**	**%**	**C.I**	**%**	**C.I**	**p**	***X***^**2**^	**Total N**
**Wealth**											
***Poorest***											
Male	51,3	44.2, 58.4	30,8	25.2, 36.9	47,2	41.2, 53.3	32,1	25.9, 39.1	0,001	27,446	1031
Female	37,1	30.7, 44.0	29,9	24.6, 35.8	39,7	34.1, 45.7	26.0	20.3, 32.6	0,009	11,667	1081
***Poorer***											
Male	44.0	37.6, 50.6	24,9	18.9, 32.2	33,8	27.1, 41.1	34,1	26.7, 42.4	0,001	15,649	770
Female	28.3	22.5, 34.9	24.0	18.3, 30.9	29,4	23.5, 36.0	27,3	20.8, 34.9	0,671	1,549	790
***Middle***											
Male	37,7	31.4, 44.3	35,2	28.2, 43.0	41.0	33.6, 48.8	32,1	24.4, 40.8	0,376	3,103	698
Female	36,1	29.4, 43.1	24.5	18.6, 31.7	32,1	25.2, 39.9	18.3	12.1, 26.8	0,002	14,387	654
***Richer***											
Male	31.1	24.9, 38.0	23.0	16.8, 30.8	31,2	23.7, 39.8	20,6	14.2, 29.1	0,084	6,661	596
Female	29.2	23.3, 36.0	14,8	9.7, 21.9	23.6	16.3, 32.9	21.6	15.1, 29.8	0,016	10,285	597
***Richest***											
Male	18.7	13.4, 25.4	25,8	18.6, 34.7	28.5	21.3, 37.0	23.4	14.9, 34.8	0,219	4,43	508
Female	13.5	9.0, 19.9	13.9	8.3, 22.3	16.9	11.1, 24.9	12.9	7.2, 22.2	0,841	0,834	480
**Total sample**	**1934**		**1754**		**1992**		**1525**				**7205**

**Table 8:** Trends in underweight among Ghanaian children by age, 0-36 Months 1993 to 2008 with 95% CI

	**1993**		**1998**		**2003**		**2008**				
**Variables**	**%**	**C.I**	**%**	**C.I**	**%**	**C.I**	**%**	**C.I**	**p**	***X***^**2**^	**Total N**
**Age**											
***0-5***											
Male	16,9	12.2, 23.0	11,8	7.6, 17.8	11.0	7.0, 16.9	9.9	6.0, 16.0	0.180	4.891	686
Female	16,9	12.3, 22.8	10.8	6.7, 16.9	7.9	4.7, 12.9	8.3	4.5, 14.6	0,026	9.269	662
***6-11***											
Male	22.8	17.2, 29.5	19.5	13.8, 26.9	26.4	20.0, 34.1	19.2	12.8, 27.7	0,375	3.110	648
Female	19.9	14.5, 26.7	17.4	12.1, 24.3	19.2	14.2, 25.5	14.4	9.3, 21.7	0,635	1.709	693
***12-23***											
Male	28.8	24.3, 33.9	23.9	19.4, 29.0	27.0	22.3, 32.2	17.8	13.2, 23.7	0,013	10.793	1298
Female	24.8	20.3, 30.1	24.0	19.5, 29.2	19,6	15.5, 24.5	16.7	12.7, 21.8	0,055	7.613	1226
***24-35***											
Male	33.9	28.7, 39.5	19.4	14.9, 24.8	22,3	17.7, 27.7	18.9	14.2, 24.7	0,001	22.797	1101
Female	27.2	22.5, 32.6	21.9	17.3, 27.5	18,6	14.4, 23.5	10.8	7.3, 15.7	0,001	21.961	1128

**Table 9:** Trends in underweight among Ghanaian children by place of residence, 0-36 Months 1993 to 2008 with 95% CI

	**1993**		**1998**		**2003**		**2008**				
	**%**	**C.I**	**%**	**C.I**	**%**	**C.I**	**%**	**C.I**	**p**	***X***^***2***^	**Total N**
**Residence**											
***Urban***											
Male	18.0	13.9, 22.9	12.3	8.4, 17.5	17,3	13.2, 22.3	14.6	10.5, 20.0	0,244	4.168	1051
Female	14.3	10.6, 19.1	15.7	11.2, 21.6	9,9	6.8, 14.2	10.4	7.2, 14.7	0.120	5.826	1014
***Rural***											
Male	30.6	27.3, 34.0	22.4	19.3, 25.8	25,3	22.0, 28.9	18.3	14.9, 22.2	0,001	26.488	2682
Female	26.4	23.3, 29.8	21.2	18.2, 24.5	21,1	18.1, 24.4	14.9	11.9, 18.5	0,001	22,331	2695

**Table 10:** Trends in underweight among Ghanaian children by region, 0-36 Months 1993 to 2008 with 95% CI

	**1993**		**1998**		**2003**		**2008**				
	**%**	**C.I**	**%**	**C.I**	**%**	**C.I**	**%**	**C.I**	**p**	***X***^***2***^	**Total N**
**Region**											
***Upper***											
Male	36.5	27.4, 46.5	29.0	22.5, 36.5	40.6	32.2, 49.5	23.6	15.9, 33.5	0,093	6,41	587
Female	33.3	25.2, 42.6	24.1	18.1, 31.2	21,6	15.4, 29.6	23.6	15.9, 33.6	0,257	4.044	567
***Middle***											
Male	24.6	19.9, 29.9	19.7	14.7, 26.0	19.0	14.6, 24.2	12.5	8.1, 18.9	0,011	11,2	947
Female	22.8	18.1, 28.3	20.6	15.5, 26.8	17,5	13.2, 22.8	12.6	8.6, 18.1	0,028	9,074	942
***South***											
Male	23,1	19.3, 27.4	17,7	14.0, 22.1	18,8	14.7, 23.8	18,2	13.8, 23.6	0,195	4,697	1358
Female	19.7	16.1, 23.9	19.2	15.6, 23.5	14,3	10.9, 18.6	11.5	8.2, 15.9	0,006	12.438	1411
***Accra***											
Male	17.0	10.7, 26.0	10.6	5.6, 19.2	13,9	7.8, 23.7	7.8	3.2, 17.8	0,278	3,852	333
Female	12.5	7.0, 21.2	11.9	6.5, 20.7	5,1	1.9, 13.0	6.5	2.7, 15.0	0,123	5.772	337
***Northern***											
Male	46.4	37.8, 55.6	36.5	27.2, 46.9	34,1	26.9, 42.1	23.9	17.0, 32.4	0,003	13,99	508
Female	35.7	26.9, 45.7	31.5	22.2, 42.5	32,6	25.6, 40.6	20.0	13.3, 29.0	0,067	7.151	452

**Table 11:** Trends in underweight among Ghanaian children by maternal education, 0-36 Months 1993 to 2008 with 95% CI

	**1993**		**1998**		**2003**		**2008**				
	**%**	**C.I**	**%**	**C.I**	**%**	**C.I**	**%**	**C.I**	**p**	***X***^**2**^	**Total N**
**Maternal education**											
***No education***											
Male	36.3	31.6, 41.3	25.5	21.3, 30.4	27,8	23.6, 32.4	20.8	16.1, 26.4	0,001	20.687	1560
Female	28.8	24.5, 33.4	24.1	19.9, 29.0	23,6	19.7, 28.0	15.3	11.2, 20.4	0,002	14,97	1545
***Primary***											
Male	22.6	19.3, 26.2	20.0	14.7, 26.6	21,4	16.2, 27.8	16.2	11.4, 22.4	0,256	4.048	1159
Female	20.3	17.1, 24.0	17.3	12.2, 23.9	14,6	10.4, 20.1	12.4	8.3, 18.1	0,057	7,531	1097
***Some high school+***											
Male	9.7	4.4, 19.9	14.0	10.6, 18.4	17,8	13.8, 22.7	14.5	10.6, 19.6	0.280	3.830	1013
Female	7.4	2.8, 18.2	17.6	13.9, 22.0	12,4	9.1, 16.7	12.2	9.0, 16.3	0,047	7.941	1066

**Table 12:** Trends in underweight among Ghanaian children by household wealth quintile, 0-36 Months 1993 to 2008 with 95% CI

	**1993**		**1998**		**2003**		**2008**				
	**%**	**C.I**	**%**	**C.I**	**%**	**C.I**	**%**	**C.I**	**p**	***X***^***2***^	**Total N**
**Wealth**											
***Poorest***											
Male	34,4	28.0, 41.4	29,3	23.9, 35.3	30,5	25.4, 36.0	20,2	15.2, 26.3	0,017	10,231	1080
Female	28,6	22.8, 35.2	25,2	20.3, 30.9	28,8	23.8, 34.4	19.0	14.2, 24.8	0,077	6,838	1113
***Poorer***											
Male	32,6	26.8, 39.0	16,8	11.8, 23.2	23,9	18.2, 30.8	18,9	13.0, 26.7	0,001	16,434	797
Female	29,3	23.5, 35.9	23,6	17.9, 30.5	17,5	13.0, 23.1	16,6	11.7, 23.1	0,007	12,084	810
***Middle***											
Male	33,9	28.0, 40.5	22,5	16.7, 29.6	22,5	16.8, 29.5	21,2	15.0, 29.2	0,009	11,491	718
Female	25,6	20.0, 32.2	15,9	11.2, 22.0	15,9	10.9, 22.5	7,7	4.0, 14.6	0,001	17,99	673
***Richer***											
Male	17,9	13.2, 24.0	14.0	9.2, 20.7	19,5	13.5, 27.2	10,5	6.1, 17.5	0,132	5,619	616
Female	18,8	13.9, 24.9	19,6	13.8, 27.2	10,8	6.5, 17.5	9,5	5.7, 15.5	0,017	10,186	615
***Richest***											
Male	12,8	8.6, 18.7	10,2	5.8, 17.3	11,2	6.8, 18.0	10,3	5.6, 18.4	0,871	0,711	522
Female	10,1	6.2, 15.8	9,8	5.4, 17.2	6,8	3.5, 12.5	8,8	4.4, 17.0	0,685	1,486	498
**Total sample**	**1970**		**1795**		**2062**		**1615**				**7442**

**Table 13:** Trends in wasting among Ghanaian children by age, 0-36 Months 1993 to 2008 with 95% CI

	**1993**		**1998**		**2003**		**2008**				
**Variables**	**%**	**C.I**	**%**	**C.I**	**%**	**C.I**	**%**	**C.I**	**p**	***X***^***2***^	**Total N**
**Age**											
***0-5***											
Male	17,5	12.6, 23.8	18.1	12.5, 25.4	13.5	8.7, 20.2	19.3	12.8, 28.1	0,534	2.188	616
Female	17,3	12.5, 23.3	11.0	6.8, 17.5	18.0	12.5, 25.3	17.8	11.6, 26.2	0,333	3.407	614
***6-11***											
Male	22.0	16.5, 28.8	23.7	17.3, 31.7	20.0	14.1, 27.7	24.1	16.8, 33.3	0.850	.799	620
Female	18.3	13.1, 25.0	21.3	15.4, 28.7	19.2	14.1, 25.5	19.7	13.6, 27.6	0,921	.492	674
***12-23***											
Male	16.7	13.1, 21.1	14.4	10.8, 18.9	11.0	8.0, 15.0	12.9	8.8, 18.6	0,171	5.009	1261
Female	13.7	10.2, 18.2	12.2	8.8, 16.5	9,1	6.2, 13.3	10.9	7.6, 15.3	0,351	3.278	1191
***24-35***											
Male	8.8	6.0, 12.8	9.0	5.9, 13.5	6,2	3.9, 9.6	4.7	2.7, 8.2	0,179	4.903	1059
Female	8.5	5.8, 12.2	5.5	3.2, 9.2	3,5	1.9, 6.4	3.5	1.7, 7.1	0,038	8.448	1096

**Table 14:** Trends in wasting among Ghanaian children by place of residence, 0-36 Months 1993 to 2008 with 95% CI

	**1993**		**1998**		**2003**		**2008**				
	**%**	**C.I**	**%**	**C.I**	**%**	**C.I**	**%**	**C.I**	**p**	***X***^**2**^	**Total N**
**Residence**											
***Urban***											
Male	11.6	8.3, 15.9	9.0	5.7, 13.9	8,5	5.6, 12.7	11.1	7.3, 16.6	0,549	2.115	1001
Female	8.9	6.0, 13.0	8.6	5.3, 13.7	9,4	6.4, 13.5	9.6	6.5, 13.9	0,981	.178	977
***Rural***											
Male	17.1	14.5, 20.1	17.2	14.4, 20.5	12,7	10.3, 15.6	14.6	11.5, 18.4	0,078	6,821	2555
Female	15.4	12.9, 18.3	12.6	10.2, 15.4	11,4	9.1, 14.2	12.6	9.8, 16.0	0,166	5,078	2598

**Table 15:** Trends in wasting among Ghanaian children by region, 0-36 Months 1993 to 2008 with 95% CI

	**1993**		**1998**		**2003**		**2008**				
	**%**	**C.I**	**%**	**C.I**	**%**	**C.I**	**%**	**C.I**	**p**	***X***^**2**^	**Total N**
**Region**											
***Upper***											
Male	26.0	18.2, 35.7	15.6	10.6, 22.3	21,3	14.4, 30.3	15.4	9.0, 25.0	0,243	4.179	546
Female	16.8	10.9, 25.2	10.9	6.9, 16.8	14,2	9.1, 21.4	18.0	11.8, 26.4	0,728	1.303	537
***Middle***											
Male	14.0	10.4, 18.6	14.7	10.2, 20.5	9,9	6.8, 14.2	11.9	7.5, 18.4	0,332	3,417	918
Female	14.7	10.9, 19.6	9.9	6.4, 15.1	9,6	6.5, 14.1	12.8	8.6, 18.7	0,247	4,141	913
***South***											
Male	12.8	9.8, 16.5	16.3	12.8, 20.8	11,1	8.0, 15.2	11.8	8.1, 16.9	0,165	5.099	1293
Female	11.2	8.4, 14.7	13.1	10.0, 16.9	11,4	8.3, 15.4	9.5	6.4, 13.8	0,508	2.326	1358
***Accra***											
Male	12.9	7.5, 21.4	6.1	2.6, 13.9	9,4	4.5, 18.6	9.5	3.9, 214	0,481	2.471	312
Female	5.7	2.4, 13.0	7.3	3.3, 15.4	6,9	3.3, 14.0	2.8	.70, 10.8	0,429	2.766	326
***Northern***											
Male	22.1	15.4, 30.7	21.2	13.9, 30.8	9,8	6.0, 15.7	20.3	13.8, 28.8	0,056	7.566	487
Female	24.2	16.6, 33.8	15.4	8.9, 25.2	12.4	8.0, 18.8	18.2	11.9, 26.7	0,125	5.732	441

**Table 16:** Trends in wasting among Ghanaian children by maternal education, 0-36 Months 1993 to 2008 with 95% CI

	**1993**		**1998**		**2003**		**2008**				
	**%**	**C.I**	**%**	**C.I**	**%**	**C.I**	**%**	**C.I**	**p**	***X***^**2**^	**Total N**
**Maternal education**											
***No education***											
Male	20.1	16.3, 24.5	20.2	16.2, 25.0	12,7	9.7, 16.5	18.8	14.0, 24.6	0,031	8,905	1477
Female	17.8	14.3, 22.0	12.4	9.2, 16.5	12,8	9.8, 16.4	15.3	11.2, 20.6	0,153	5.267	1486
***Primary***											
Male	13.4	10.8, 16.6	13.4	9.0, 19.6	11,6	7.7, 17.1	10.2	6.3, 16.0	0,671	1,548	1106
Female	11.0	8.6, 14.1	12.4	8.1, 18.6	11,3	7.6, 16.6	8.4	5.2, 13.4	0,681	1,505	1066
***Some high school+***											
Male	6.8	2.6, 16.7	11.1	8.0, 15.2	9,7	6.8, 13.5	11.2	7.6, 16.1	0,704	1.407	972
Female	7.7	2.9, 18.8	10.6	7.8, 14.4	8,2	5.5, 11.9	10.2	7.3, 14.4	0,643	1.671	1022

**Table 17:** Trends in wasting among Ghanaian children by household wealth quintile, 0-36 Months 1993 to 2008 with 95% CI

	**1993**		**1998**		**2003**		**2008**				
	**%**	**C.I**	**%**	**C.I**	**%**	**C.I**	**%**	**C.I**	**p**	***X***^**2**^	**Total N**
**Wealth**											
***Poorest***											
Male	14,8	10.3, 20.7	24.0	18.9, 30.1	11,6	8.3, 16.0	13,9	9.6, 19.8	0,003	14,036	1016
Female	17,6	12.9, 23.5	14,5	10.6, 19.5	15,8	11.9, 20.7	15,9	11.6, 21.5	0,844	0,823	1075
***Poorer***											
Male	16,4	12.0, 21.9	13,4	8.9, 19.8	16.0	11.4, 22.1	15,1	9.6, 22.9	0,848	0,806	759
Female	18.0	13.3, 24.0	15,3	10.6, 21.6	7,8	4.8, 12.5	12,5	8.1, 18.8	0,021	9,712	777
***Middle***											
Male	21,5	16.5, 27.5	15,6	10.7, 22.1	9,6	6.0, 14.9	19,6	13.2, 27.9	0.010	11,348	692
Female	14.6	10.3, 20.3	8.0	4.8, 13.0	8.0	4.8, 13.1	11,5	6.5, 19.6	0.100	6,251	646
***Richer***											
Male	14,4	10.0, 20.1	11,4	7.0, 18.0	11,6	7.0, 18.5	9,2	5.2, 15.9	0,523	2,245	592
Female	10.3	6.7, 15.4	9,4	5.5, 15.7	9.1	5.2, 15.6	8.1	4.4, 14.3	0,891	0,624	597
***Richest***											
Male	9.0	5.5, 14.4	5,7	2.6, 11.8	6,5	3.2, 12.7	6.0	2.5, 13.8	0,638	1,693	497
Female	5,8	3.0, 10.8	8,7	4.5, 16.1	11,6	7.0, 18.5	6,4	2.9, 13.7	0,285	3,788	480
**Total sample**	**1913**		**1742**		**1957**		**1519**				**7131**

In the age-specific data for stunting shown in Table 2, decline occurred primarily in the 24–35 month age group for both males (F (1, 2171) = 15.42, p < .001) and females (F (1, 2171) = 9.91, p ≤ .002). The slope of the trends did not differ by sex.

**Figure 1:**
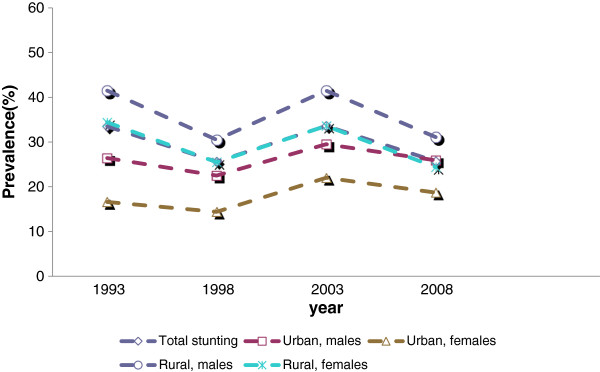
Stunting trends by rural/urban.

In the analysis of urban/rural place of residence, shown in Table 3, there was a stable and not statistically significant trend for urban males and females and rural females, while the declining trend for rural males was statistically significant (F (1 ,5203) = 7.23, p ≤ .007). The decline for boys contributed to an apparent narrowing of the urban/rural gap in stunting, as shown in Figure 1.

Decomposing the analysis of stunting by regions (Table 4) revealed that the declining trends were significant for males only in the Northern region (F (1,931) = 3.95, p ≤ .047) and Middle region (F (1, 1843) = 7.19, p ≤ .007), and not significant for females in any of the regions.

**Figure 2:**
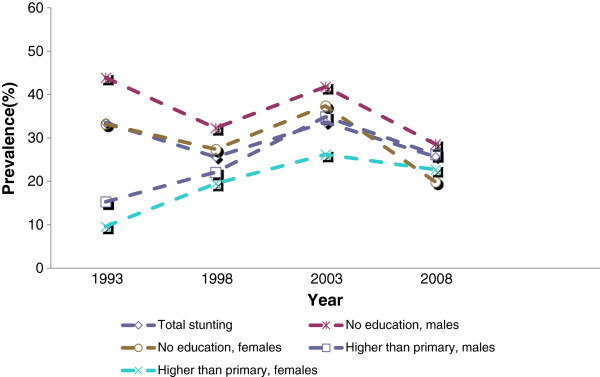
Stunting trends by maternal education.

The analyses of stunting by maternal education (Table 5) reveal a narrowing in the gap in nutritional status between various groups stratified by maternal education. This is due in part to a significantly worsening trend in stunting among males having mothers with higher than primary education (F (1, 2004) = 3.92, p ≤ .048) and among females having mothers with higher than primary education (F (1, 2004) = 4.34, p ≤ .037). This is illustrated in Figure 2. Stratifying the analysis by urban/rural place of residence, Table 6 shows significantly declining trends in stunting among males of mothers with no education in the rural sample (F (1, 2570) = 7.07, P ≤ .008), while females of mothers with more than primary education exhibit worsening trends (F (1, 1103) = 5.01, P ≤ .025). Males also exhibit statistical significant increasing trends in the urban sample (F (1, 900) = 5.80, P ≤ .016).

In the analyses of stunting by level of household wealth (WI), shown in Table 7, statistically significant declines were observed for females in the poorest wealth quintile (F (1, 2111) = 4.39, P ≤ .036) and for males in the poorer wealth quintile (F (1, 1559) = 5.99, p ≤ .014).

### Trends in underweight (weight-for-age)

Table 9 shows the underweight prevalence estimates by age and residence. The declining trend for females in the 0–5 month age group was significant (F (1, 1347) = 7.47, p ≤ .006), as was the case in the 12–23 month age group for males (F (1, 2523) = 8.48, p ≤ .004) and for females (F (1, 2523) =5.03, p ≤ .025). The declines in underweight were also statistically significant in the 24–25 month age group, both for males (F (1, 2228) = 19.90, p ≤ .001) and females (F (1, 2228) = 12.88, p ≤ .001). The stable to worsening trends in underweight for males and females in the 6–11 month age group did not achieve statistical significance.

**Figure 3:**
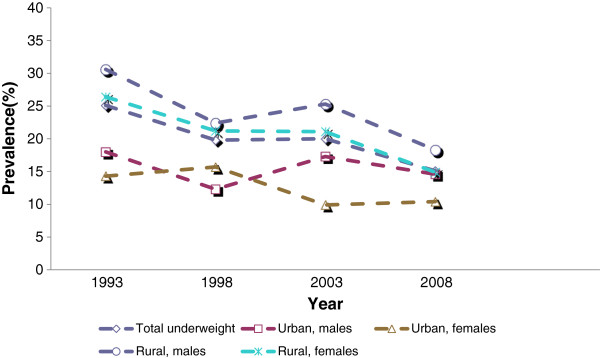
Underweight trends by rural/urban.

In the analyses of underweight by rural/urban place of residence, the declining trends were significant for rural males (F (1, 5376) = 24.22, p ≤ .001) and rural females (F (1, 5376) = 14.22, p ≤ .001), while the declining trends for urban males and females were not significant. The result was an apparent narrowing in the urban/rural underweight gap, as shown in Figure 3.

In the analyses by region (Table 10), significant declining trends were observed in the Middle region for males (F (1, 1888) = 11.99, p ≤ .001) and females (F (1, 1888) = 5.71, p ≤ .017) and in the Northern region for males (F (1, 959) = 17.66, p ≤ .001). The trends in the other regions did not reach statistical significance.

**Figure 4:**
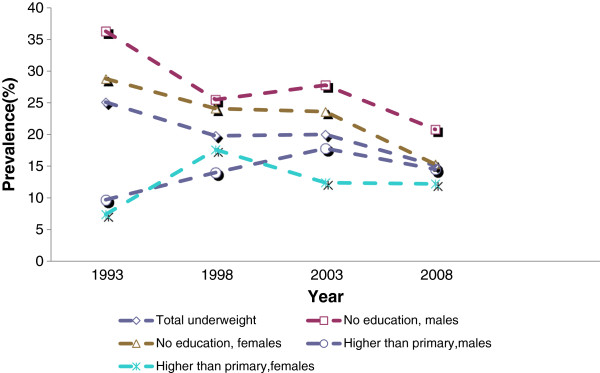
Underweight trends by maternal education.

In the analysis of underweight by maternal education (Table 11), there was a distinct narrowing of the gap in underweight prevalence between children whose mothers have no education and those whose mothers have higher than primary education, as shown in Figure 4. This was due in part to significant declining trends for males having mothers with no education (F (1, 3104) = 18.95, p ≤ .001) and for females having mothers with no education (F (1, 3104) =10.79, p ≤ .001).

In the analysis of underweight by household wealth (Table 12), statistically significant declining trends were observed in the poorest wealth quintile for males (F (1, 2192) = 9.18, p ≤ .002), in the poorer wealth quintiles for males (F (1, 1606 ) = 11.24, p ≤ .001) and females (F (1, 1606) = 5.09, p ≤ .024), in the middle quintiles for males (F (1,1390) = 10.66, p ≤ .001) and females (F (1, 1390) = 8.50, p ≤ .004), and in the richer quintile for females (F (1, 1230) = 4.88, p ≤ .027).

### Trends in wasting (weight-for-height)

Tables 13, 14, 15, 16 and 17 show prevalence estimates for wasting. There was a significant declining trend for males in the 12–23 month age group (F (1, 2451) = 4.31, p ≤ .038) and in the 24–35 age group (F (1, 2451) = 9.58, p ≤ .002). None of the other trends had significant slopes.

## Discussion

This study examined long-term trends in child malnutrition among children less than three years of age in Ghana, by region, by rural–urban setting and by socio-economic and demographic characteristics. The findings at the national level show that overall child malnutrition is significantly decreasing. Stunting, the effect of chronic under-nutrition, declined from 33.5% in 1993 to 25.7% (23% reduction) by 2008. This is in sharp contrast with the complete stagnation of stunting trends in the West African Sub-region as a whole [2]. Thus, Sub-regional trends cannot be used to estimate trends in countries within a region, at least not in the case of Ghana. Yet, given the current trend, the rate of decline in stunting is not likely to move Ghana to the level where it will meet the WHO target of a 40% reduction by 2025 [35].

Child underweight has also declined significantly in Ghana, from 25.1% in 1993 to 15.1% in 2008, a 60% reduction. Thus, by 2008 Ghana had already achieved the Millennium Development Goal (MDGs) target of halving 1990 levels of underweight by 2015. This achievement could be attributed to significant reduction in extreme poverty in Ghana over the past two decades. For instance, overall poverty declined from 51.7% in 1991 to 28.5% in 2006 [36,37]. Similarly, the number of people living below the extreme poverty line in the country decreased by more than half in the last two decades. Total food production also increased significantly in Ghana during this period [36], essential for an increase in the availability of food to the population. Another possible explanation of the achievement made by Ghana with regard to underweight reduction is the introduction of the National Plan of Action on Nutrition (NPAN) by the Government of Ghana in 1995 [38], which aims to combat malnutrition in the country. The NPAN is multimodal, including the Baby–Friendly Health Facility Initiative, the Community Based Nutrition Behavior Change Communication (BCC) strategy, mother-to-mother support groups for promoting optimal breastfeeding and complementary feeding practices, and community-based growth promotion projects, are a few of the NPAN’s key elements.

The situation for wasting is not as positive as it is for stunting and underweight. Even though Ghana has made some progress in the reduction of wasting between 1993 and 2008, the national prevalence remains high at 12.4% for children less than 36 months, a level the WHO classifies as requiring urgent response [39]. Wasting, which is usually the result of acute significant food shortage and/or disease, is a strong predictor of mortality among children under-five years [18]. Hence, for Ghana to be on track to achieve MDG 4, there is a need for re-doubled effort to address childhood wasting.

A number of important departures from the national trends raise cause for concern. The situation at the regional level is a complete departure from the national level. Our results show that the Middle region is the only region that exhibited significantly declining trends in stunting and underweight both for both males and females, revealing that not all regions enjoyed the declining trends seen in the national-level analyses. In terms of prevalence, the Northern and Upper regions have the highest levels of malnutrition, and this has also been documented in previous studies [5,14]. The disproportionate burden of malnutrition on children in the Northern part of Ghana may be attributable the North’s high rate of poverty [36,37]. While the reduction in overall poverty at the national level between 1992 and 2006 was remarkable, the three Northern regions did not record significant poverty reduction [36,37]. Additionally, over 70% of people whose incomes are below the poverty line can be found in the Savannah areas [36,37]. The observed improvement in poverty statistics at the national level has not been equitably distributed. As poverty is a strong driver of malnutrition [40], sustained high poverty rates in the North translate into sustained malnutrition in the children of the North. Poverty and malnutrition are mediated by inadequate food consumption and infectious disease. Insufficient availability of food at household level due to poverty and/or improper feeding practices places children at an elevated risk of malnutrition, both directly and by malnutrition’s influence on a child’s susceptibility to disease and infection, and immune system dysfunction [41].

It is clear from the above discussion that the overall positive national trends mask important variation at the sub-national level. This demonstrates that sub-national level analyses are important for identifying regions and social groups that need better support and interventions to improve the malnutrition situation. Further, there is evidence that even the regional level prevalence data may mask differences in other important segments of the country [42].

Our data suggest that differentials in child malnutrition by place of residence have substantially narrowed over time in Ghana, consistent with other studies [15]. This trend in urban–rural differentials is primarily due to static trends in urban malnutrition coupled with rural improvement. A number of factors might have contributed to the improving trends in rural areas. One such factor is the significant increase in food production in Ghana between 1992 and 2008 [36,37]. Since much of the food consumed in Ghana is produced in the rural areas, it is plausible that food supplies became increasingly available to rural households during this period, and when food is available in the household, it is assumed that children will be well fed. Another possible explanation for the urban–rural differentials is that poverty is viewed in Ghana as an overwhelming rural phenomenon [43]. Therefore, most antipoverty initiatives are directed at the rural population. However, the emphasis on the alleviation of rural poverty has led to a degree of neglect of the problems of urban poverty and urban food insecurity. In fact, urban poverty and associated health problems are growing in Ghana [44,45]. The introduction of nutrition rehabilitation services and the Supplementary Feeding and Health and Nutrition Education Programmes [38], could also explain the narrowing gap between rural and urban settings. These programmes are targeted at deprived communities, mostly rural communities. These explanations are sensible, but remain merely speculative, because the possible contributory factors identified above were not measured in the surveys that provided the data for this study.

The gap in malnutrition by maternal education level has significantly narrowed in Ghana, to the extent that malnutrition rates for children of mothers who have no education are indistinguishable from children of educated ones in 2008. These differences in trends are partly due to increasing malnutrition in children of mothers with more than primary education. Further analysis stratified by place of residence was undertaken as part of this study, to examine the possibility that the effect of education is confounded by place of residence. This analysis did not provide evidence of such confounding.

The findings above are unexpected because it is assumed that mothers who have high education will be more empowered to be able to take decisions on the type of nutrition and care the child should receive. Educated mothers could also be in a better position to make informed nutritional decisions that buck unhealthy cultural norms about child feeding [46]. The puzzling failure of maternal education to have the expected effect on child nutritional status in Ghana could be due to structural factors. One such factor is intervention targeting. Most nutrition and antipoverty interventions in Ghana are targeted at the population considered disadvantaged, in this case, the non-educated and rural population [38]. This places the so-called advantaged groups, who also have pressing needs, at a disadvantage. The re-coding of the education variable could also be a plausible explanation. Our data have few people who have higher education and as a result, secondary education was collapsed with higher education. This could mask the importance of higher maternal education as a protective factor. Additionally, it is important to note that Ghana has very high unemployment rates [36,37], and as a result most mothers with secondary education are unlikely to find a decent job to earn a living, and consequently may find it difficult to provide for the basic needs of their children.

Our data show that child malnutrition trends in the poorest wealth quintiles are decreasing while trends in the richest quintiles remain static. Even though this suggests that children in the poorest quintiles are getting better over time, malnutrition remains the bane of the poor in Ghana. The results show that malnutrition prevalence among children in the poorest wealth quintile is as much as twice that of the richest quintile. These findings corroborate earlier studies in Ghana using DHS data, which found that children in the poorest households are more than twice at risk of being malnourished compared to their counterparts in the richest households [5,23]. However, the declining trends among the poorest quintiles implies that the halving of people living below the extreme poverty line, and the significant increase in food production in Ghana between 1991 and 2008 [36,37] have helped to close the malnutrition equity gap.

The child’s age is an important factor in level of risk for malnutrition. Malnutrition is more prevalent in older than in younger children [5-7]. In our data, the oldest children (24–35 months) have the highest levels of malnutrition, and the youngest age group (0–5 months) has the lowest levels. With regard to time trends, the older children exhibited significant improvement compared to the younger ones in the case of stunting. However, both younger and older age groups exhibited significant declining trends in the case of underweight. The higher proportion of malnutrition among older children could be due to inappropriate child feeding practices and/or increased morbidity, while the declining malnutrition trends may be explained by systematic improvement in the availability and quality of food [36,37] and other care practices during this period. This is an empirical issue that could be addressed using the GDHS data on child care and feeding; however such analyses are beyond the scope of this paper.

There are limitations in the analysis reported in this paper. The core data of this study come from the anthropometry measurements and the birth date data used to calculate the growth variables. As reported in our results, data of sufficient quality was obtained from between 87 and 90 percent of eligible children depending on survey year. There are a myriad reasons why useable anthropometry data might be missing, discussed in detail by Pullum [47]. These include poor technical work by data collectors, faulty equipment, sick or uncooperative children, refusal by the mother or another household member, the child being away from the household during the data collection window, and data entry errors at the time of data collection and/or in the transfer of data to analyzable data files, among other reasons. While missing data is of inevitable concern in survey research, what would be of greater concern would be a systematic pattern over the survey years wherein various reasons for missing data increased or decreased in prevalence from survey to survey. We do not have detailed enough missing data analyses from the four surveys to evaluate the seriousness of this potential source of bias. There is evidence that poor birth data was a minor cause of missing growth data in all the survey.

Besides missing data, the validity of the malnutrition trends reported in this paper may be compromised due to method variation in determining which children were eligible for measurement. In 1993 and 1998 surveys, anthropometric measurements were restricted to children born to the women who were interviewed. Children were excluded if their mothers were not in the household, if their mothers were not eligible for the individual interview, or the mother did not complete an interview. The methodology changed in the 2003 and 2008 surveys, and children who slept in the household the night before data collection were eligible regardless of the interview status of their mother. As a result, orphans and children whose mothers were away were excluded in 1993 and 1998 and included in 2003 and 2008. This would pose a validity issue if orphans, for example, are more likely to suffer malnourishment than non-orphans in the same household. At least one study has examined this issue, comparing South African orphans and non-orphans in the same households [48]. There were no significant differences between the two groups on health outcomes. Nevertheless, the change in the sampling protocol in 2003 is a source of concern for trend analyses such as this paper reports.

## Conclusions

The analysis at the national level shows that child malnutrition is significantly declining in Ghana. However, the aggregate national trends mask the fact that not all segments of the population benefit from improvement of the same magnitude, as our findings by various geographic and demographic characteristics reveal. There is a need for policies that address the specific constraints of households left out of progress so that children from all segments of the country benefit. Additionally, the study also found that the widened gap between rural and urban settings in Ghana is closing. While child malnutrition is progressively decreasing in the rural areas, it remains static in the urban settings. This contributed greatly to narrowing the rural/urban child malnutrition gap. In addition, the malnutrition trends by maternal education in Ghana have narrowed to the extent that the differences between the educated and non-educated are not easily distinguishable. A perplexing finding of this study is the increasing trends of childhood stunting among children of mothers who have higher than primary education, in sharp contrast with existing literature. This anomaly needs further investigation.

## Competing interest

The authors declare that they have no competing interest.

## Authors’ contributions

DAA and MBM designed the study. DAA performed the data analysis, interpreted the results and drafted the manuscript. MBM supervised all parts of the study and contributed to the methodology and revision of the manuscript. AL contributed to the planning of the study and revision of the manuscript. All authors read and approved the final version of the manuscript.

## Pre-publication history

The pre-publication history for this paper can be accessed here:

http://www.biomedcentral.com/1471-2458/13/960/prepub

## References

[B1] de Onis M BLossner M Borghi E Morris SS Frongillo EA Methodology for estimating regional and global trends of child malnutrition Int J Epidemiol 2004 33 1260 1270 10.1093/ije/dyh202 15542535

[B2] de Onis M Blossner M Borghi E Prevalence and trends of stunting among pre-school children, 1990–2020 Public Health Nutr 2010 10.1017/S1368980011001315 21752311

[B3] de Onis M Edward A Blossner M Is malnutrition declining? An analysis of changes in levels of child malnutrition since 1980 Bull World Health Organ 2000 78 1222 1233 11100617 PMC2560621

[B4] WHO World Health Organization global data base on child growth and malnutrition 2011 Geneva cited 2012 24.10]; Available from: http://www.who.int/nutgrowthdb/database/countries/gha/en/

[B5] de Poel V Hosseinpoor RA Jehu-Appiah C Vega J Speybroeck N Malnutrition and the disproportionate burden on the poor: the case of Ghana Int J Equity Health 2007 6 21 10.1186/1475-9276-6-21 PMC2245943 18045499

[B6] Omilola B Paterns and trends of child and maternal nutrition inequalities in Nigeria 2010 Washington DC: International Food Policy Research Institute

[B7] Ponguo R Ezzati M Salomon AJ Household and community socioeconomic and environmental determinants of child nutritional status in Cameroon BMC Public Health 2005 98 6 10.1186/1471-2458-6-98 PMC1523206 16618370

[B8] Girma W Timotiows G Determinants of nutritional status of women and children in Ethiopia 2002 ORC Macro: Calverton, Maryland

[B9] de Onis M Onyango AW Borghi E Garza C Yang H Comparison of the world health organization (WHO) child growth standards and the national center for health statistics/WHO international growth reference: implications for child health programmes Public Health Nutr 2006 9 7 942 947 17010261 10.1017/phn20062005

[B10] de Onis M New WHO child growth standards catch on Bull World Health Organ 2011 89 250 251 21479088 10.2471/BLT.11.040411 PMC3066529

[B11] Kuate-Defo B Areal and socioeconomic differentials in infant and child mortality in Cameroon Soc Sci Med 1996 42 399 420 10.1016/0277-9536(95)00107-7 8658234

[B12] Smith LC Ruel MT Ndiaye A Why is child malnutrition lower in urban than rural areas? Evidence from 36 developing countries World Dev 2005 33 8 1285 1305 10.1016/j.worlddev.2005.03.002

[B13] Bosu WK Nsorwah-Nuamah N Ward PM A profile of health inequalities in Ghana 2000 Accra: Ghana Ministry of Health

[B14] Smith LC Marie T Ndiaye A Why is child malnutrition lower in urban than rural areas? Evidence from 36 developing countries 2004 Washington DC: International Food Policy Research Institute Contract No. FCNDP 176

[B15] Fotso J-C Urban–rural differentials in child malnutrition: trends and socioeconomic correlates in sub-Saharan Africa Health Place 2007 13 205 223 10.1016/j.healthplace.2006.01.004 16563851

[B16] Ruel MT Garnett JL Morris SS Urban challenges to nutrition security: a review of food security, health and care in the cities. Food consumption and Nutrition Discussion Paper 1998 Washington DC: International Food Policy Research Institute Contract No.: 51

[B17] Haddad L Marie T Ruel Garrett JL Are urban poverty and undernutrition growing? Some newly assembled evidence World Dev 1999 27 11 1891 1904 10.1016/S0305-750X(99)00093-5

[B18] UNICEF Progress for children: A report card on nutrition 2006 New York

[B19] Gayle H Dibley M Marks J Trowbridge F Malnutrition in the first two years of life. The contribution of low birth weight to population estimates in the United States Am J Dis Child 1987 141 4 531 534 3578166 10.1001/archpedi.1987.04460050073034

[B20] Borrell C Muntaner C Benach J Artazcoz L Social class and self-reported health status among men and women: what is the role of work organisation, household material standards and household labour? Soc Sci Med 2004 58 10 1869 1887 10.1016/S0277-9536(03)00408-8 15020005

[B21] Dancer D Rammohan A Maternal autonomy and child nutrition: evidence from rural Nepal Indian Growth Dev Rev 2009 2 1 18 38 10.1108/17538250910953444

[B22] Hong R Banta JE Betancourt J Relationship between household wealth inequality and chronic childhood under-nutrition in Bangladesh Int J Equity Health 2006 15 5 10.1186/1475-9276-5-15 PMC1702347 17147798

[B23] Hong R Effects of economic inequality on chronic childhood undernutrition in Ghana Public Health Nutr 2005 4 10 372 378 10.1017/S1368980007226035 17362533

[B24] Asenso-Okyere WK Asante FA Nube M Understanding the health and nutritional status of children in Ghana Agric Economies 1997 17 59 74 10.1016/S0169-5150(97)00011-X

[B25] Frimpong JA Ponguo R Does economic growth improve child health? understanding discordant trends in malnutrition indicators during the economic growth in Ghana 2008 Population Association of America: New Orleans, LA

[B26] Burchi F Child nutrition in Mozambique in 2003: the role of mother's schooling and nutrition knowledge Econ Hum Biol 2010 8 331 345 10.1016/j.ehb.2010.05.010 20646971

[B27] MEASURE DHScited 2012 17.9]; Available from: http://www.measuredhs.com/data/available-datasets.cfm

[B28] WHO Multicenter Growth Reference Study Group WHO Child Growth Standards: length/height-for-age, weight-for-age, weight-for-length, weight-for-height and body mass index-for-age: methods and development 2006 Geneva, Switzerland: World Health Organization

[B29] Ghana Statistical Service (GSS), Ghana Health Service (GHS) Demographic and Health Survey 2008 2009 Ghana: Accra, Ghana: GSS, GHS, and ICF Macro

[B30] Ghana, Statistical, Service (GSS), Noguchi Memorial Institute of Medical Research ORC Macro Ghana Demographic and Health Survey 2003 2004 Calverton, Maryland: GSS, NMIMR, and ORC Macro. Ghana

[B31] Ghana Statistical Service (GSS), Macro International Inc Ghana Demographic and Health Survey 1998 1999 Calverton, Maryland: GSS and MI. Ghana

[B32] Ghana Statistical Service (GSS), Macro International Inc. (MI) Ghana Demographic and Health Survey 1993 1994 Calverton, Maryland: GSS and MI. Ghana

[B33] WHO Child Growth Standards SPSS Syntax File[cited 2012 12.9]; Available from: http://english6.net/w/who-child-growth-standards-spss-syntax-file-(igrowup-e2534-pdf.pdf

[B34] Bosu WK Nsorwah-Nuamah N Ward PM A profile of health inequalities in Ghana 2000 Ghana Ministry of Health

[B35] WHO Proposed global targets for maternal, infant and young child nutrition 2012 Geneva: WHO

[B36] Commission NDP UNSiG: 2008 Ghana Millennium Development Goals 2010 Ghana: NDPC

[B37] Commission NDP UNSiG. Achieving the MDGs with equity in Ghana: unmasking the issues behind the averages 2012 Ghana: National Development Planning Commission

[B38] Government of Ghana National Plan of Action on Food and Nutrition 1995–2000 1995 Ghana

[B39] WHO Global database on child growth and malnutrition WHO [cited 2012 9.09]; Available from: http://www.who.int/nutgrowthdb/about/introduction/en/index5.html

[B40] Asian Development Bank Improving child nutrition in Asia. Nutrition and Development, Volume No. 3 2001 Manila: ADB

[B41] Haggerty P Pande R Sanchez A Nutrition and health status of young children and their mothers in Mali: Findings from the 1995/96 Mali Demographic and Health Survey 1998 Macro International, Inc: Calverton, Maryland

[B42] Morris S Levin CE Amar-Klemesu M Maxwell D Ruel MT Does geographic targeting of nutrition interventions make sense in cities? evidence from Abidjan and Accra World Dev 1999 27 2011 2019 10.1016/S0305-750X(99)00098-4

[B43] Boateng Oti E Ewusi K Kanbur R McKay A A poverty profile for Ghana J Afr Econ 1992 1 1 25 58

[B44] Maxwell D Levin C Armar-Klemesu M Ruel M Morris S Ahiadeke C Urban Livelihoods and Food and Nutrition Security in Greater Accra, Ghana 2000 WASHINGTON, D.C: International Food Policy Research Institute

[B45] Coulomb H McKay A An assessment of trends in poverty in Ghana:1988–92. PSP Discussion Paper 81 1995 Washington, D.C: World Bank

[B46] Kandala N-B Madungu TP Emina JB Nzita KPD Cappuccio FP Malnutrition among children under the age of five in the Democratic Republic of Congo (DRC): does geographic location matter? BMC Public Health 2011 261 11 10.1186/1471-2458-11-261 PMC3111378 21518428

[B47] Pullum TW An Assessment of the Quality of Data on Health and Nutrition in the DHS Surveys, 1993–2003. Methodological Reports No. 6 2008 Calverton, Maryland, USA: Macro International Inc.

[B48] Parikh A DeSilva MB Cakwe TQ Simon JL Skalicky A Zhuwau T Exploring the Cinderella myth: intrahousehold differences in child wellbeing between orphans and non-orphans in Amajuba District, South Africa AIDS 2007 21 suppl 7 S95 S103 10.1097/01.aids.0000300540.12849.86 18040170

